# Molecular studies on ARX gene in syndromic and non-syndromic mental retardation

**DOI:** 10.1186/1755-8166-7-S1-P75

**Published:** 2014-01-21

**Authors:** Gayatri Kulkarni, Suvidya Ranade

**Affiliations:** 1 Department of Chemistry, University of Pune, Pune, Maharashtra, India

**Keywords:** Mental retardation (MR), ARX, exon, mutation

## Background

Mental retardation (MR) is frequently the result of genetic mutations. Syndromic mental retardation is intellectual deficits associated with other medical and behavioral signs and symptoms. Non-syndromic mental retardation refers to intellectual deficits that appear without other abnormalities. The newly identified ARX (Aristaless related homeobox) gene consists of five exons and encodes 562 amino acid proteins and is thought to regulate brain development. Mutations in the ARX gene are associated with a diverse spectrum of phenotypes ranging from severe developmental abnormalities of the brain to syndromic and nonsyndromic forms of X-linked mental retardation (XLMR) syndromes that can be associated with normal or abnormal brain morphology. Mutations in the human ARX gene are the major cause of developmental and neurological disorders.

## Material and methods

The present study was focused on screening of exon 2 of ARX gene in Indian families with mental retardation in order to obtain the relative prevalence of ARX mutations. Method adapted in present study was amplification of target exon by using polymerase chain reaction, qualitative conformation of amplicons by agarose gel electrophoresis and their use for conformation sensitive gel electrophoresis to find heteroduplex formation which is followed by sequencing.

## Conclusion

In present study we have found insertion mutation at genomic position 25031668, this change leads to loss of homeobox, OAR domains of ARX protein. The other mutation obtained was substitution of C to A/G is observed and does not affect protein functioning.

